# HIV infection in Xi’an, China: epidemic characterization, risk factors to false positives and potential utility of the sample-to-cutoff index to identify true positives using Architect HIV Ag/Ab combo

**DOI:** 10.1186/s13756-019-0463-0

**Published:** 2019-01-11

**Authors:** Linchuan Wang, Yao Xiao, Xu-Dong Tian, Jin-xiong Ruan, Wei Chen, Yan Yu

**Affiliations:** 1 Clinical Laboratory of the First Affiliated Hospital of Xi’an Jiaotong University, Yan Ta Road No 277, Xi’an, Shaanxi Province China; 2 Inspection Department of Hong-Hui Hospital, Xi’an Jiaotong University College of Medicine, Nan Guo Road No 76, Xi’an, Shaanxi Province China

**Keywords:** HIV epidemic, False positives, Architect HIV ag/ab combo

## Abstract

**Background:**

In China, although tremendous efforts has been made, the HIV/AIDS is still not controlled.

**Objectives:**

The study was carried out to determine the epidemic of HIV infection in Xi’an, analyse false positives (FP) risk factors and potential utility of sample-to-cutoff index to identify true positives using Architect HIV Ag/Ab Combo.

**Methods:**

A retrospective review for HIV screening by Architect HIV Ag/Ab Combo was performed in a teaching hospital in Xi’an between 2015 and 2016. The prevalence of HIV, positive predictive value (PPV) at different cut-off indexices (COI) were calculated. The epidemic of infections and risk factors for FP results were investigated.

**Results:**

In the study, the HIV prevalence and FP rate of Architect HIV Ag/Ab Combo were 0.076 and 46.08%, respectively. The Han Chinese, males and people aged < 40 years accounted for the majority of infections (98.29, 76.07 and 73.5%, respectively). 85.47% of the infections were transmitted through sexual contact (35.04% of male homosexual and 50.43% of heterosexual). COI at 1–10, 10–30 and ≥ 30, the PPVs were 0, 50 and 100%, respectively. The independent risk factors for FP, i.e., pregnancy and malignancy had a statistically significant association with FP (*p* < 0.05), and age had a very strong statistically significant association with FP (*p* < 0.001).

**Conclusions:**

In Xi’an, sexual contact was the most important transmission mode for HIV, and the infections were predominantly identified in Han Chinese, males, young and middle-aged people. For Architect HIV Ag/Ab Combo, it can achieve 100% of PPV with COI ≥30, and the age was strongly statistically associated with FP.

## Background

**Fig. 1:**
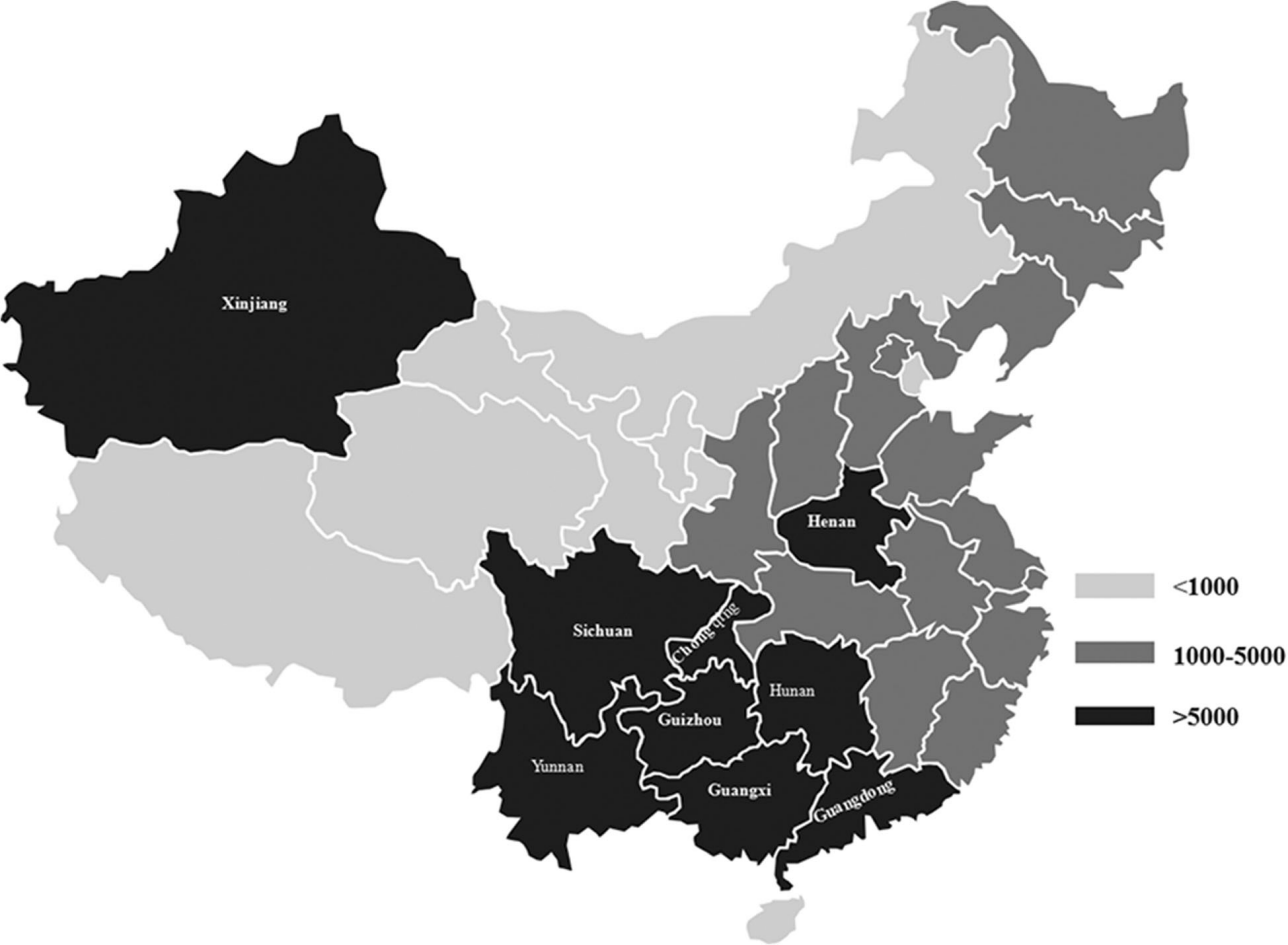
The geographic distribution of newly reported HIV/AIDS cases among people aged > 15 years in 2015 in Mainland China. Among the 114,656 new infections, 9 provinces with more than 5000, 14 provinces with 1000–5000 and 8 provinces with less than 1000, respectively, accounted for 66.5, 30.1 and 3.4% of the cases in 2015 in mainland China

The transmission of HIV/AIDS in China has undergone entry (1985–1988), expansion (1989–1993) and rapidly increasing phase (1994-present) [1, 2]. Since one AIDS case from an Argentine tourist in Beijing and four HIV infections from hemophiliac patients undergoing treatment with Factor VIII in Zhejiang Province were identified in 1985 [1–4], the spread rate of HIV in China is alarming. In 1994, HIV/AIDS cases had been reported in 22 provinces [3], and since 1998, a nation-wide spread had been observed in China [1, 2, 5]. In 2015, 577,423 people were living with HIV/AIDS in mainland China [4, 6], and among people aged > 15 years, more than 5000 new cases had been reported in 9 provinces, and 1000–5000 new cases had been reported in 14 provinces (Fig. 1) [7]. Although quite a few prevention policies and tremendous efforts for HIV/AIDS transmission have been made and performed in China, i.e., “ban on imported blood products” [2], blood donors must be tested for HIV since 1995 [1], “Action Plan on HIV/AIDS Prevention” [2, 8], “Methadone Maintenance Treatment (MMT) program” [8–10], and “Four Free One Care” [2, 9], the HIV/AIDS epidemic in China is still not controlled or slowed down.

New and recent HIV infections are more infectious than chronic infections [11–14], thus early identification of HIV infection is crucial to prevent HIV transmission [15]. The algorithm requires a sequence of tests to identify HIV infection for the repeatedly reactive subjects. Currently, the 4th^-^generation assay is widely applied to screen HIV in China. Simultaneously detecting the p24 antigen and HIV-1/2 antibodies, the 4th^-^generation assay is more sensitive than the 3rd-generation assay with approximately 2 weeks window period [16–19]. But nonspecific reactivity [20–24] was often observed because the assay combines two different test principles in one assay. Since the 4th^-^generation assay was used as the initial HIV test in our hospital, the incidence of false positives was more frequent than the use of 3rd-generation assay before. HIV false positives may cause inappropriate anxiety to patients or even undesirable consequences. Especially for the sample which was only reactive to the 4th^-^generation assay, we could not differentiate early seroconversion from false-positive.

The present study was carried out to investigate the epidemic of HIV infection in Xi’an, to analyze the positive predictive value (PPV) at different COI, and to identify the risk factors for FP using Architect HIV Ag/Ab Combo.

## Methods

### Study population

This study was conducted between January 1, 2015 and October 31, 2016 in the First Affiliated Hospital of Xi’an Jiaotong University, which is the largest hospital in Northwest China. It is a 2541-bed teaching hospital with about 3 millions outpatients annually. During the study period, a total of 154,005 patients underwent HIV screening (77,525 males [50.34%]; median age 52 years [range:2–89 years]). All repeatedly reactive patients were included in the study. The data in the study were available from the LIS and HIS of the First Affiliated Hospital of Xi’an Jiaotong University and Xi’an, Center for Disease Control and Prevention (CDC), Shaanxi Province, China.

### The flow chart for identifying HIV infection

A 4th^-^generation kit, Architect HIV Ag/Ab Combo (Abbott Diagnostics, Abbott Park, IL) and a 3rd-generation EIA kit, XinChuang HIV-1/2Ab (InTec, INC, XiaMen, FuJian, China) were used as the first and retesting assays, respectively. COI or S/CO ≥1 was defined as reactive, and COI or S/CO < 1 was defined as non-.reactive.

According to the Chinese CDC guideline, the ID card and telephone should be recorded in the “REPORT OF HIV SCREENING TESTING” and send one copy to the CDC if the result of individual is repeatedly reactive to screening test, and the specimen should be submitted for confirmation (WB, nucleic acid or p24 antigen tests). If the confirmatory test is not positive, follow-up at the 1st, 3rd and 6th month is required to exclude HIV infection. Currently, the nucleic acid and p24 antigen tests are not applied at CDC in Xi’an for HIV confirmation. Thus, for the repeatedly reactive subjects by HIV Ag/Ab Combo, the identifying algorithm used in our hospital was—WB should be conducted if it was also reactive to HIV-1/2Ab. For the only HIV Ag/Ab Combo-reactive subjects, we contacted them through the recorded telephone at the 2nd, 4th week, 3rd and 6th month to carry out HIV-1/2Ab test. Once HIV-1/2Ab was reactive, then WB should be performed. If the initial WB was negative or indeterminate, the subjects were required to perform four WB tests (at week 2, week 4, month 3 and month 6) by Xi’an CDC.

### Western blotting

Western blotting HIV1/2 BLOT 2.2 (MP Biomedicals, Singapore) was conducted at the Xi’an CDC. The WB results were interpreted according to the Chinese CDC criteria: Positive— the presence of at least two bands, including two *env* bands, or one *env* band plus one *gag* band; Indeterminate—reactivity to any of the bands but not compatible with the criteria for a positive interpretation; Negative—the absence of any of the specific bands.

### Statistical analysis

Statistical analyses were performed by SPSS13.0 (serial number 5026743; SPSS Inc., Chicago, Illinois, USA), and WB positive was the standard for HIV infection diagnosis in the study. The Mann-Whitney U-test was used for continuous variables (medians of the age and COI) comparison of between true-positive (TP) and false-positive (FP) patients, because they were not .normal distribution. Categorical variables were compared using the Fisher’s exact test. The risk factors of false positives were evaluated using logistic regression analysis. A *p*-value < 0.05 was considered to be statistically significant.

## Results

### Epidemic of true-positive and false-positive populations

**Fig. 2:**
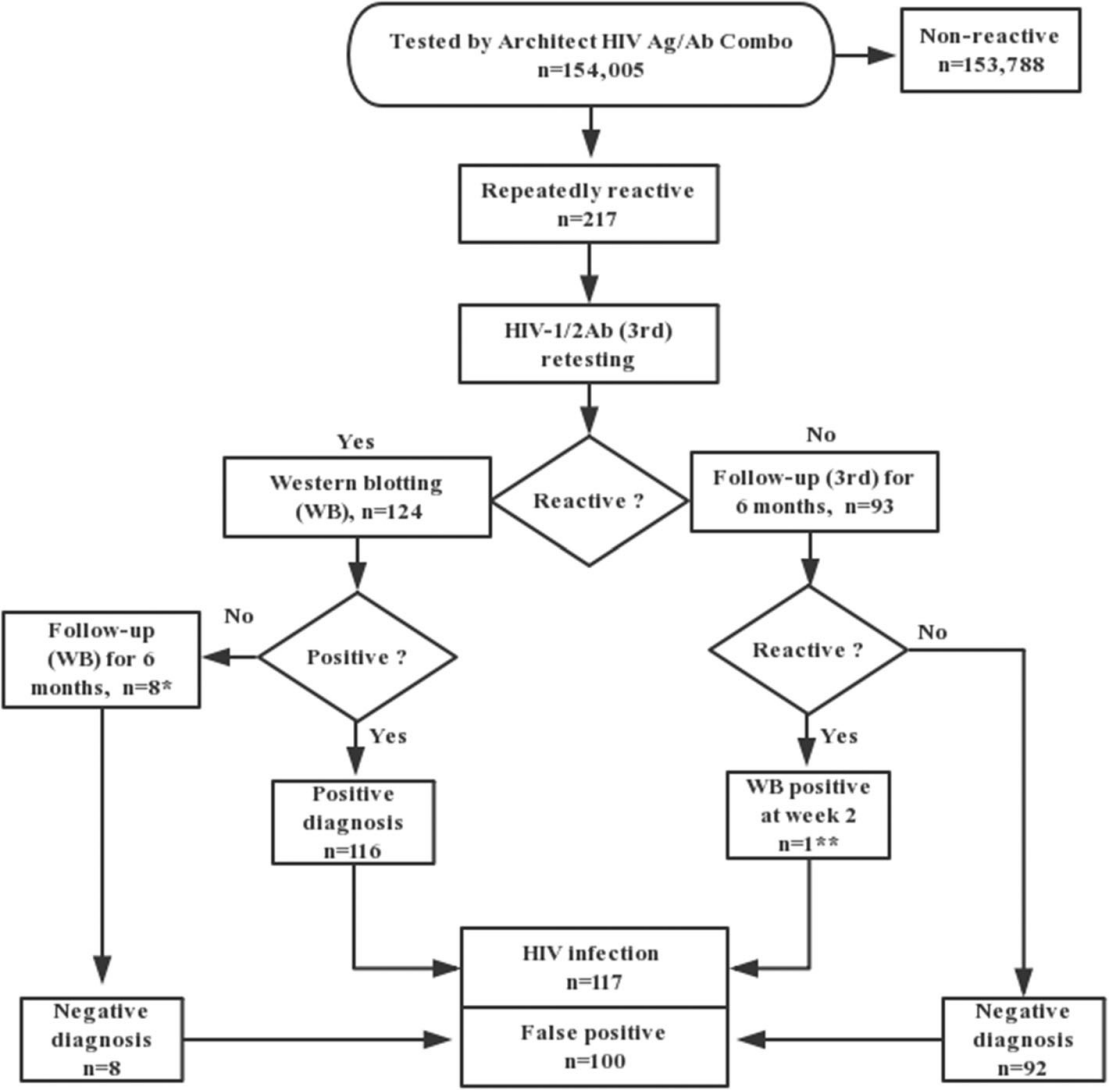
The flow chart for identifying the 117 HIV infections and 100 false positives. * 7 cases were WB negative and one case was WB indeterminate (p24 band). ** one case was reactive to HIV-1/2Ab test and WB positive (gp120/gp160 and p24 bands) at the 2nd week of follow-up

During the study period, a total of 217 patients who were repeatedly reactive by ARCHITECT HIV Ag/Ab Combo were included. Among them, HIV-1/2Ab reactive and non-reactive numbers were 124 and 93. According to the identifying algorithm used in our hospital, 116 and 8 of the 124 cases were diagnosed as positive by the initial WB and negative by four follow-up WB tests, respectively. For the 93 cases with only HIV Ag/Ab Combo reactive results, 92 cases were non-reactive to four follow-up tests by HIV-1/2Ab assay, one case was reactive to HIV-1/2Ab assay and WB positive (gp120/gp160 and p24 bands) at the 2nd week of follow-up. Overall, according to the results of WB and six months follow-up, 117 and 100 of the 217 cases were classified as TP and FP for HIV infection, respectively, Fig. 2. The median age of the FP population was 52.5 years (range 20–84 years; males [*n* = 61]; females [*n* = 39]). The Han Chinese, hospitalized patients and married cases accounted for the majority of the FP population (99, 91 and 92%, respectively). The distributions of < 40, 40–60 and ≥ 60 years for the FP population were 20, 31 and 49%, respectively, Table 1.

**Table 1:** Characteristics of true-positive and false-positive population

Characteristics	TP population *n* = 117	FP population *n* = 100	*p*-value
Sex			
Male	89 (76.07%)	61 (61%)	0.019
Female	28 (23.93%)	39 (39%)	
Age, median (IQR), years	30 (26–41)	52.5 (45–63.5)	0.000
─ 40	86 (73.5%)	20 (20%)	0.000
40–60	27 (23.08%)	31 (31%)	0.219
≥ 60	4 (3.42%)	49 (49%)	0.000
Ethnicity			
Han	115 (98.29%)	99 (99%)	>0.05
Minority	2 (1.71%)	1	
Source of patients			
Outpatients	106 (90.6%)	9 (9%)	0.000
Hospitalized patients	11 (9.4%)	91 (91%)	
Marital status			
Unmarried	52 (44.44%)	8 (8%)	0.000
Married	65 (55.56%)	92 (92%)	
Transmission modes			
Male homosexual contact	41 (35.04%)	∕	
Heterosexual contact	59 (50.43%)	∕	
Injecting drug	6 (5.13%)	∕	
Unknown	11 (9.4%)	∕	
Job			
College students	22 (18.8%)	∕	
Unemployed/informal employees	34 (29.06%)	∕	
Formal employees	50 (42.74%)	∕	
Peasants	2 (1.71%)	∕	
Unknown	9 (7.69%)	∕	
COI, median (IQR)	470.53 (273.99–628.21)	3.21 (1.82–8.58)	0.000
Clinical symptoms			
Flu-like			
Fever	12	1	
Painful pharynx and larynx	2	0	
Swelling of tonsil	4	0	
Swelling of lymph nodes	5	0	
Total	23 (19.66%)	1 (1%)	0.000
Skin diseases			
Rashes	6	1	
Genital herpes	4	0	
Genital condyloma	4	0	
Perianal condyloma	3	0	
Perianal abscess	10	0	
Total	27 (23.08%)	1 (1%)	0.000

The TP population comprised 89 males and 28 females with a median age of 30 years (range: 17–72 years). Hans Chinese, outpatients and married cases accounted for the majority of infections (98.29, 90.6 and 55.56%, respectively). The constituent ratios of the transmission modes were 85.47% for sexual contact (male homosexual contact, 35.04%; heterosexual contact, 50.43%), 5.13% for injecting drug and 9.4% for unknown. Among the infections, one juveniles case (17 years) and 6 older cases (≥60 years) were observed. The age-specific distributions of infections were 73.5% for < 40 years, 23.08% for 40–60 years and 3.42% for ≥60 years. Of the 117 infections, 22 (18.8%) were college students, 34 (29.06%) were unemployed/informal employees, 50 (42.74%) were formal employees, 2 (1.71%) were peasants and 9 (7.69%) were unknown, Table 1.

Compared to the FP population, the TP population was more likely to be younger (30 vs 52.5, *p* = 0.000), unmarried (44.44% vs 8%, *p* = 0.000) and from outpatient (90.6% vs 9%, *p* = 0.000). The proportion of males in the TP population was significantly higher than that in the FP population (76.07% vs 61%, *p* = 0.019). Flu-like clinical symptoms (*n* = 23) and skin diseases (*n* = 27) were presented in 42.74% (50/117) of the TP patients, i.e., fever (*n* = 12), painful pharynx and larynx (n = 2), swelling of the tonsils (*n* = 4) and lymph nodes (*n* = 5), rashes (*n* = 6), genital herpes (n = 4) and condyloma (n = 4), perianal condyloma (*n* = 3) and abscess (*n* = 10), Table 1.

### Consistency and PPV at different COI values

**Fig. 3:**
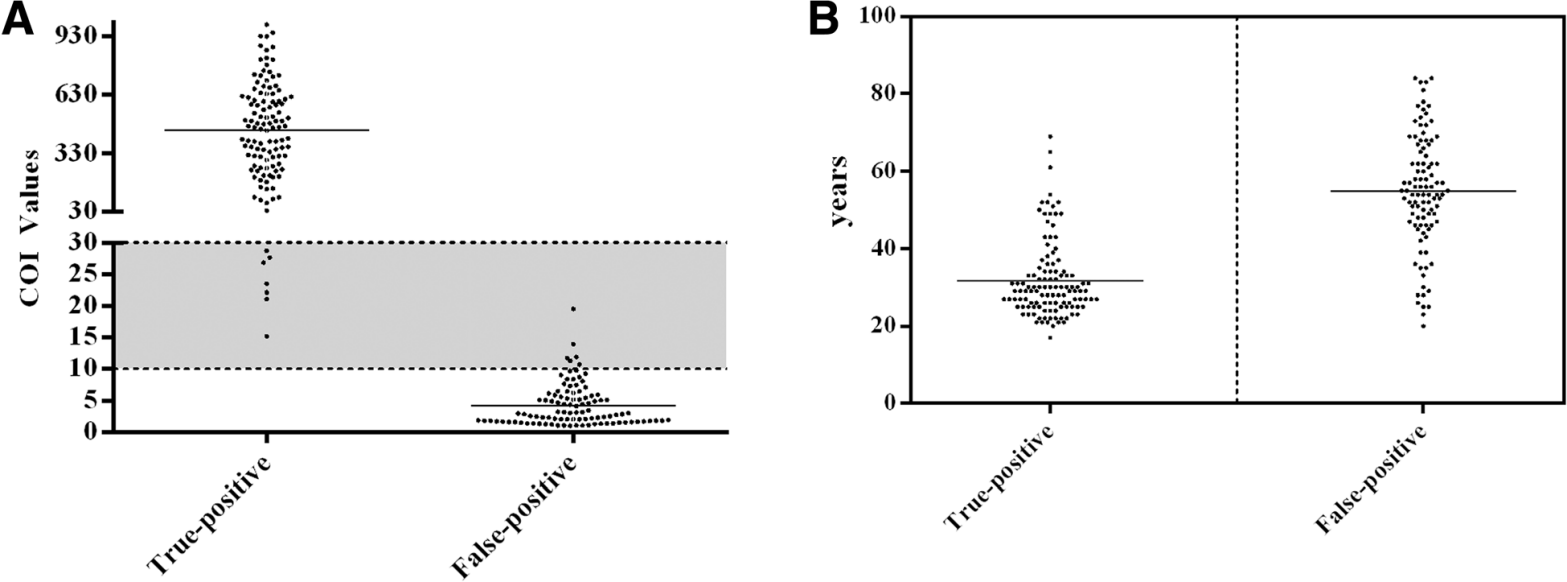
The comparison between HIV true-positive and false-positive group of **a** the COI values and **b** age

Of the 217 repeatedly reactive subjects, the COI for the FP population was significantly lower than that for the TP population (3.21 vs 470.53, *p* = 0.000), (Fig. 3a and Table 1). They were divided into low (L: 1 ≤ COI < 10), medium (M: 10 ≤ COI < 30) and high (H: COI ≥30) groups. In the L group (range, 1.01–9.71), only 2 of 93 cases were reactive to HIV-1/2Ab retesting, and all of the 93 cases were classified as FP. In the M group (range, 10.72–27.66), 12 of 14 cases were retest-reactive to HIV-1/2Ab and 6 cases were WB positive, but one case who was only 4th^-^generation-reactive had seroconversion for HIV-1/2Ab and WB at the 2nd week of follow-up. All of the 110 cases in the H group (range, 37.65–1049.84) were HIV-1/2Ab reactive and WB positive. Overall, consistency (4th^-^generation and HIV-1/2Ab assays) and PPV were 2.15% and 0 for the L group, 85.71 and 50% for the M group and 100, 100% for the H group, respectively. In total, 116 of 124 cases with consistent results (reactive to both 4th^-^generation and HIV-1/2Ab assays) were TP (PPV: 93.55%), and 1 of 93 with inconsistent result (only reactive to 4th^-^generation assay) was TP (PPV: 1.08%), Table 2.

**Table 2:** Consistency and PPV at different COI values for HIV screening

First-test-reactive (Ag/Ab Combo)		Retesting by 3rd-generation			PPV (%)
Groups (COI Values)	N0.	Reactive (TP)	Non-reactive (TP)	Consistency(%)	
L (1.00–10.00)	93	2 (0)	91 (0)	2.15	0
M (10.00–30.00)	14	12 (6)	2 (1)	85.71	50
H (≥30.00)	110	110 (110)	0	100	100
Total	217	124 (116)	93 (1)	57.14	53.92

Note: *TP* was true positive

### Analysis of risk factors for HIV false positives

**Fig. 4:**
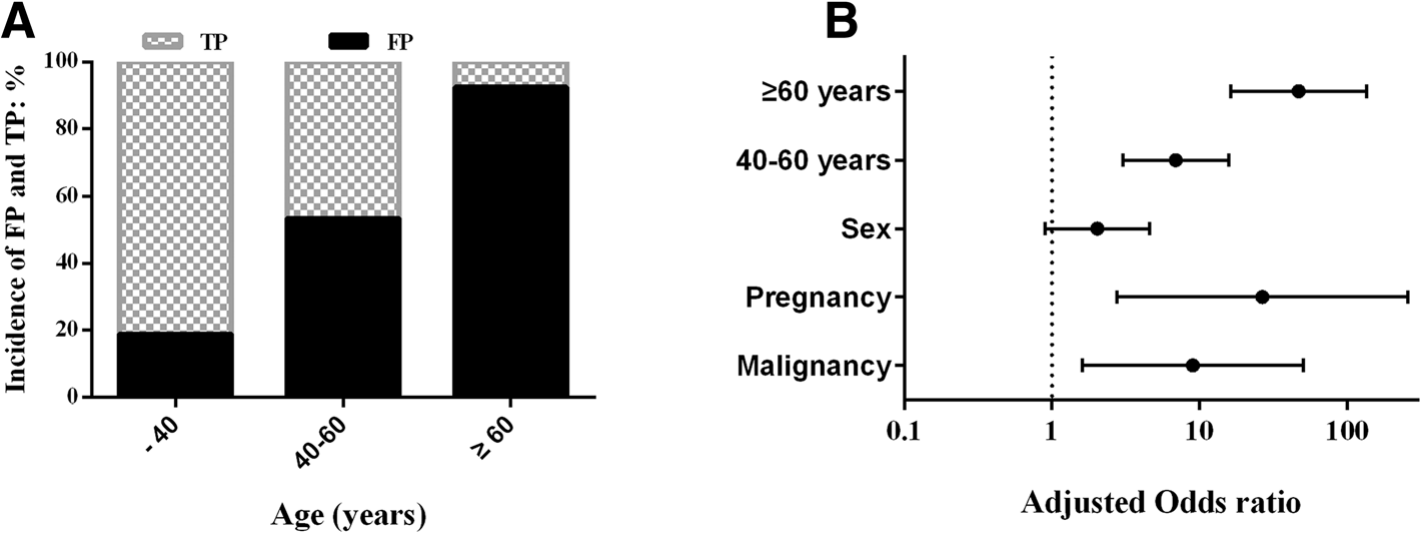
The incidence of TP and FP at age-specific distributions for repeatedly reactive subjects (**a**), and (**b**) the adjusted odds ratio and 95% confidence intervals of risk factors for FP by multivariate analysis

In our study populations, the prevalence of HIV was 0.076% (117/154,005). Although the ARCHITECT HIV Ag/Ab Combo had a specificity of 99.94% (153,788/153888) for HIV diagnosis, in fact, of the 217 repeatedly reactive subjects by the assay, the FP rate was 46.08% (100/217). The FP patients were significantly older than the TP patients, (52.5 vs 30, *p* = 0.000), Fig. 3b. With increased age, the incidence of TP decreased, but that of FP increased, Fig. 4a.

Univariable analysis showed that the sex, age, malignancy and pregnancy were significantly associated with FP, *P <* 0.05. Multivariable analysis was conducted to control for the effects of confounding variables. In this analysis, a dependent variable was defined as the presence of FP and independent variables were intrinsic factors, i.e., sex, age, ethnicity and comorbidity. The final logistic regression analysis showed that age, malignancy and pregnancy were independent risk factors for FP. Among the risk factors, malignancy and pregnancy had a statistically significant association with FP (*p* < 0.05), and the age (≥40 years) had a very strong statistically significant association with FP (*p* < 0.001). Patients who were malignancy, pregnancy, at 40–60 and ≥ 60 years were 9, 26.58, 6.9 and 46.85 times, respectively, more likely to be tested as FP compared to the control patients (Table 3 and Fig. 4b).

**Table 3:** Binary Logistic Analysis for the risk factors of HIV false positives

Characteristics	False-positive		Crude			Adjusted		
	Yes	No	OR	95%CI	*p*-value	OR	95%CI	*p*-value
Sex								
Male	61	89	1			1		
Female	39	28	2.03	1.13–3.65	0.017*	2.03	0.9–4.57	0.09
Age, years								
─ 40	20	86	1			1		
40–60	31	27	5.44	2.64–11.22	0.000	6.9	3.02–15.78	0.000***
≥ 60	49	4	36.25	13.65–96.24	0.000	46.85	16.28–134.81	0.000***
Ethnicity								
Han	99	115	1			1		
Minority	1	2	1.72	0.15–19.28	0.659	0.71	0.06–9.09	0.794
Comorbidity								
Renal diseases								
No	95	115	1			1		
Yes	5	2	3.03	0.57–15.95	0.192	1.47	0.13–17.28	0.761
HBV infection								
No	95	98	1			1		
Yes	5	2	3.03	0.57–15.95	0.192	1.77	0.13–24.56	0.670
Malignancy								
No	83	115	1			1		
Yes	17	2	11.78	2.65–52.37	0.001***	9	1.61–50.4	0.012*
Pregnancy								
No	94	116	1			1		
Yes	6	1	6.11	0.70–53.16	0.101	26.58	2.75–256.6	0.005*
Autoimmune diseases								
No	98	117	1			1		
Yes	2	0	4.83	0.53–43.97	0.162	9.35	0.57–152.74	0.117

Notes: *Statistically significant association, *P* < 0.05; ***very strong statistically significant association, *P* < 0.001

## Discussion

The prevalence of HIV in our study populations was 0.076%, which was close to other studies in China [25–27]. Men who have sex with men (MSM), female sex workers (FSWs) and injecting drug users (IDUs) are the three most-at-risk populations in China [10]. With the effective implementation of needle exchange^2^ and MMT programs [8–10, 28], now, sexual contact especially through MSM has become the very significant transmission mode in China [29, 30]. In our study, the main characteristics for the HIV infection epidemic in Xi’an were: (i) sexual contact as the predominant transmission mode, with MSM and heterosexual transmission accounting for 35.04 and 50.43%, respectively of the cases; (ii) the majority of infections were Han Chinese (98.29%) and aged < 40 years (73.5%), and; (iii) the HIV-infected population has recently shifted from the at-risk population to the general population. Among the 117 infections in the study, one case was 17 years old, 6 cases were ≥ 60 years, and 14 infections were college students.

Previous reports [21, 22] have indicated that the PPV of 4th-generation assay was poor. In our study, although it seemed that the specificity (99.94%) of ARCHITECT HIV Ag/Ab Combo was excellent and was also consistent with other reports [31, 32]. Moreover, of the 217 repeatedly reactive cases by the assay, 46.08% were found to be FP. We found that the FP rate decreased as the test values increased [14, 33]. For ARCHITECT HIV Ag/Ab Combo, the FP rate was 100% at 1–10 of COI, but the FP rate decreased to 0 when the COI was ≥30. A variety of intrinsic factors for FP were investigated in the study. Univariable analysis showed that sex, age, malignancy and pregnancy were significantly associated with FP (*p* < 0.05). Multivariable analysis indicated that age, malignancy and pregnancy were the independent risk factors for FP. There was a statistically significant association for malignancy and pregnancy with FP (*p* < 0.05), and a very strong statistically significant association for age with FP (*p* < 0.001). Patients aged ≥60 years are 46.85 times more likely to be tested as FP compared to the control patients (< 40 years).

If test results are repeatedly reactive to the 4th-generation assay, a WB should be performed in according with the routine algorithm of the CDC guideline, and specimen with WB negative or indeterminate result should undergo nucleic acid testing (NAT). However, NAT and p24 antigen tests are not currently applied at the CDC and hospitals in Xi’an for the diagnosis of HIV infection. Considering that WB is a confirmatory test, a WB-negative result is often regarded as exclusion of HIV infection by the patient and clinician in China, and the incorrect understand and treatment could likely lead to serious consequences for the early infections. In theory, only 4th-generation-reactive implies that the antibodies to HIV are absent or insufficient in the blood, and a subsequent WB is not essential. Thus, a 4th-generation assay as the first test to shorten the window period, a 3rd-generation retesting for HIV-1/2Ab as the supplemental test to determine performing WB or not, and the follow-up protocols to shorten diagnosis of HIV infection and exclude HIV infection were used in our hospital. In the study, if WB was conducted for the 93 cases who were only reactive to HIV Ag/Ab Combo, they may be tested as WB negative. Although 92 of the cases were FP, one case had seroconversion at the 2nd week of follow-up.

## Conclusions

In the study, the HIV infection epidemic and the shortcomings of the current HIV testing algorithm used in Xi’an, China, were investigated and the potential utility of the sample-to-cutoff index to discriminate FP from TP was conducted. Overall, in Xi’an, sexual contact was the most important mode for HIV transmission, and the infections were predominantly found in Han Chinese, males, young and middle-aged people. The HIV-infected population has recently shifted from the at-risk population to the general population. HIV infection in juveniles, older people and college students should be more concerned. With regard to the Architect HIV Ag/Ab Combo, COI at 1–10 and ≥ 30 can be used as a reliable value for FP and TP, respectively, and being aged ≥60 years was the most significant risk factor for FP.

## Abbreviations

AIDS: Acquired Immune Deficiency Syndrome
CDC: Center for Disease Control and Prevention
COI: Cutoff index
FP: False positives
FSWs: Female sex workers
HIV: Human immunodeficiency virus
IDUs: Injecting drug users
MMT: Methadone Maintenance Treatment
MSM: Men who have sex with men
TP: True positives
WB: Western blotting
